# The emerging role of radiation therapists in the contouring of organs at risk in radiotherapy: analysis of inter-observer variability with radiation oncologists for the chest and upper abdomen

**DOI:** 10.3332/ecancer.2020.996

**Published:** 2020-01-06

**Authors:** Simona Arculeo, Eleonora Miglietta, Fabrizio Nava, Anna Morra, Maria Cristina Leonardi, Stefania Comi, Delia Ciardo, Massimo Sarra Fiore, Marianna Alessandra Gerardi, Matteo Pepa, Simone Giovanni Gugliandolo, Lorenzo Livi, Roberto Orecchia, Barbara Alicja Jereczek-Fossa, Samantha Dicuonzo

**Affiliations:** 1 Division of Radiation Oncology, European Institute of Oncology IRCCS (IEO), Via Ripamonti 435, 20141 Milan, Italy; 2 Department of Oncology and Hemato-oncology, University of Milan, Via Festa del Perdono 7, 20122, Milan, Italy; 3 Unit of Medical Physics, European Institute of Oncology IRCCS (IEO), 20141 Milan, Italy; 4 Radiation Oncology Unit, Oncology Department, Azienda Ospedaliero-Universitaria Careggi, University of Florence, Largo Piero Palagi 1, 50139 Florence, Italy; 5 Scientific Directorate, European Institute of Oncology IRCCS (IEO), 20141 Milan, Italy

**Keywords:** contouring, inter-observer variability, organs at risk (OAR), radiation therapist

## Abstract

**Aims:**

To compare the contouring of organs at risk (OAR) between a clinical specialist radiation therapist (CSRT) and radiation oncologists (ROs) with different levels of expertise (senior–SRO, junior–JRO, fellow–FRO).

**Methods:**

On ten planning computed tomography (CT) image sets of patients undergoing breast radiotherapy (RT), the observers independently contoured the contralateral breast, heart, left anterior descending artery (LAD), oesophagus, kidney, liver, spinal cord, stomach and trachea. The CSRT was instructed by the JRO e SRO. The inter-observer variability of contoured volumes was measured using the Dice similarity coefficient (DSC) (threshold of ≥ 0.7 for good concordance) and the centre of mass distance (CMD). The analysis of variance (ANOVA) was performed and a *p*-value < 0.01 was considered statistically significant.

**Results:**

Good overlaps (DSC > 0.7) were obtained for all OARs, except for LAD (DSC = 0.34 ± 0.17, mean ± standard deviation) and oesophagus (DSC = 0.66 ± 0.06, mean ± SD). The mean CMD < 1 cm was achieved for all the OARs, but spinal cord (CMD = 1.22 cm). By pairing the observers, mean DSC > 0.7 and mean CMD < 1 cm were achieved in all cases. The best overlaps were seen for the pairs JRO-CSRT(DSC = 0.82; CMD = 0.49 cm) and SRO-JRO (DSC = 0.80; CMD = 0.51 cm).

**Conclusions:**

Overall, good concordance was found for all the observers. Despite the short training in contouring, CSRT obtained good concordance with his tutor (JRO). Great variability was seen in contouring the LAD, due to its difficult visualization and identification of CT scans without contrast.

## Introduction

Conformal radiotherapy (RT), based on three-dimensional technique and, more recently, on intensity-modulated RT (IMRT), is widely used as a modern approach to postoperative treatments for breast cancer. Conformal RT allows for dosimetric optimization and increases organs at risk (OAR) sparing, which represents major goals of treatment planning [1], and more tailored dose distributions to the target volume are obtained with IMRT [2]. The accuracy of dose delivery is provided by means of image-guided RT, which ensures the reproducibility of patient positioning at each treatment fraction.

Conformal RT is based on complex procedures which start from the accurate delineation of the clinical target volume (CTV) and OARs [3, 4]. The contouring task is a fundamental pre-requisite for high-quality plans and a source of potential inaccuracy. Great efforts are spent to lower the inter-observer variability by using atlases, auto-contouring software, and attending live contouring workshops [5].

Furthermore, in the RT planning process, volume delineation is the most time-consuming task for clinicians as it relies more and more on registration and interpretation of multimodality imaging [6]. The inter-observer variability on contouring might have implications in terms of dosimetric distribution and clinical outcomes and has been the subject of several studies [7, 8].

In clinical practice, the contouring of target volumes and OARs is performed by radiation oncologists (ROs) [9]. In the literature, most of the contouring studies are focused on assessing the variability among physicians (ROs and radiologists) [10–14]. Since in recent years, the role of clinical specialist radiation therapists (CSRTs) has gained ground in RT planning, treatment delivery and patient care [15], appropriately trained CSRTs might substitute ROs in contouring OARs [10, 16, 17], with the aim of improving the workflow.

The purpose of the current study is to quantify the inter-observer variability in OARs delineation in the chest and upper abdomen between ROs, with different levels of expertise, and a trained CSRT.

## Materials and methods

This study was carried out within the research project entitled ‘Adjuvant radiation treatments with IMRT and/or hypofractionated schedules for breast cancer’, notified to the European Institute of Oncology Ethics Committee (May 26, 2016, Milan, Italy). All patients gave written informed consent for the treatment and anonymous use of their data for educational and research purposes. The non-contrast 2.5-mm-thick computed tomography (CT) of the chest and upper abdomen of ten patients receiving RT to the breast for breast cancer (BC) using TomoTherapy® Hi-Art System (Tomotherapy Inc., Madison, WI), acquired in the supine position, were used for OARs delineation. OARs were contoured using the treatment planning system RayStation® (version 6.1.1.2, RaySearch Laboratories AB, Stockholm, Sweden).

The observers were given written documentation regarding delineation, according to clinical daily practice. In addition, the CSRT was presented the delineation software and CT scans. The group of observers included a senior RO (SRO) with more than 10-year experience, a junior RO (JRO) with 2-year experience, a second-year training fellow RO (FRO) with a 3-month experience in the thoracic district contouring, and a CSRT with 500-hour working experience in RT department. The CSRT received a 100-hour training course in contouring by the JRO under the supervision of the SRO. In total, the CSRT contoured approximately 110 OARs from ten patients during the training period.

The contoured OARs were contralateral breast, heart, oesophagus, ipsilateral kidney, left anterior descending artery (LAD), liver, spinal cord, stomach, and trachea. Lung was not considered because the contouring was automatically generated. The delineation of the heart (starting inferior to the left pulmonary artery to the apex, including the fatty tissue within the pericardium and excluding the superior vena cava), LAD (from the left main coronary artery, running between the left atrium and ventricle and extending to the apex), oesophagus (from the cricoid cartilage to the gastroesophageal junction), spinal cord (including all the spaces within the bony canal from the cricoid cartilage to the bottom of L2), and contralateral breast (breast gland, excluding pectoralis and intercostal muscles and rib plane) was based on the Atlas for breast volumes contouring developed by the Italian Association of Radiation Oncology (AIRO) and available online [18]. The kidney and liver were contoured with the exclusion of the hilum, and the stomach was contoured from the cardiac region to the pyloric antrum and trachea from the bottom of the larynx to the carina. The observers independently contoured the OARs.

The variations between observers were quantified by means of the Dice similarity coefficient (DSC) and the centre of mass distance (CMD).

The DSC was used to evaluate the overlap between the delineated contours. DSC represents the intersection between two observers’ contours, for the same OAR, normalized to their intersection. Since the DSC varies from 0 (no overlap) to 1 (perfect overlap), a threshold value of 0.7 was considered as the minimum level of good concordance [19]. The CMD represents the distance between the centre of mass of two delineated contours for the same OAR and was measured in centimetres. DSC and CMD were calculated for each OAR by RayStation®.

The distributions of the evaluated parameters were assessed using the analysis of variance (ANOVA); the significance *p*-value threshold was set to 0.01. The analysis was performed using Matlab (v. R2014a, The Mathworks Inc.).

For the purpose of the study, we compared the inter-observer variability of contouring of each OAR and, in every couple of observers, we evaluated the consistency of contouring with each other considering all the OARs as a whole.

The observers were paired into sets of two: JRO versus FRO, FRO versus CSRT, SRO versus CSRT, SRO versus JRO, SRO versus FRO and JRO versus CSRT.

## Results

All CT scans were acquired in the period between April and June 2017. In total, 360 contours were generated by the four observers.

### Inter-observer variability

**Figure 1.**
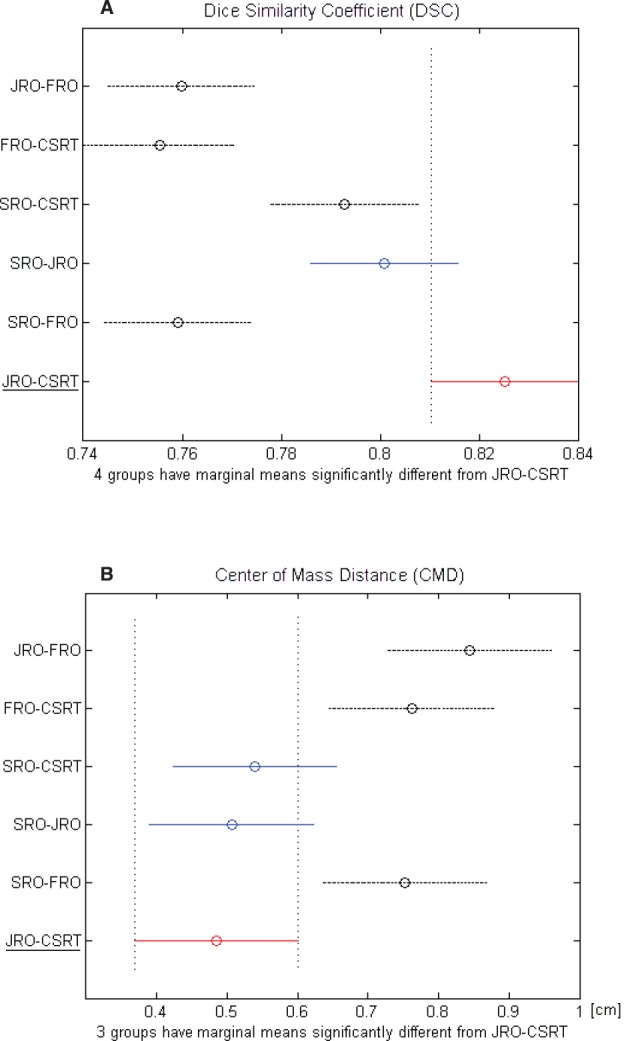
(A) DCA and (B) CMD of each couple of observers. The black dashed line indicates the couple that is significantly different from the red-underlined reference couple.

Table 1 shows the inter-observer variability of the OAR delineation. The mean DSC between all the couples of observers was > 0.7 and the mean CMD was < 1 cm. The best concordance was found between CSRT and JRO (DSC = 0.83 cm and CMD = 0.49 cm), who were taken as the reference pair. Comparing each other sets of observers with the reference pair, no statistical difference was found with the pair SRO-JRO with regard to both parameters (DSC = 0.80 cm and CMD = 0.51 cm) (Figure 1a) and with the pair SRO-CSRT with respect to CMD alone (Figure 1b). The FRO reported the highest variability when compared to the other observers for both parameters.

### OAR contour analysis

**Figure 2.**
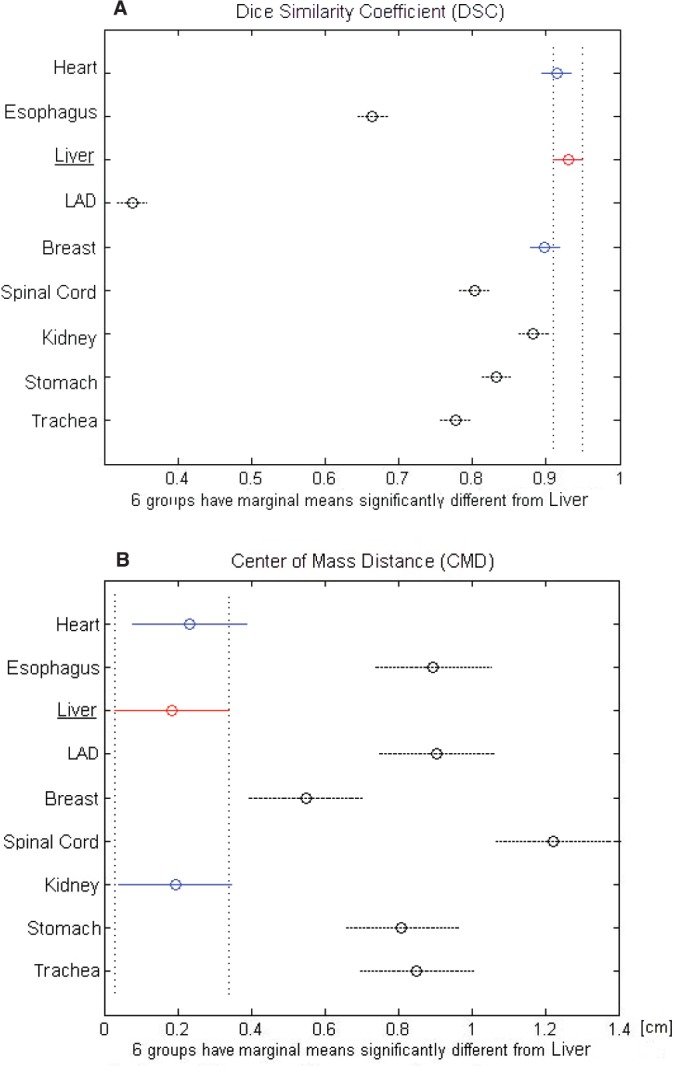
(A) DCA and (B) CMD for each analysed organ at risk (OAR). The black dashed line indicates the OARs that are significantly different from the red-underlined reference (liver).

The largest variability in contouring affected the LAD, which presented a mean DSC of 0.34 ± 0.17, followed by the oesophagus with a mean DSC of 0.66 ± 0.06 (Table 2). Regarding the CMD, a poor value (>1 cm) was reported only for the spinal cord (CMD = 1.22 ± 1.00 cm). All other OARs showed a mean DSC > 0.7. In particular, stomach, spinal cord, and trachea showed a mean DSC of about 0.80, whereas heart, liver, breast, and kidney had a mean DSC in the range of 0.88–0.93 (Table 2). The best overlapping was obtained for the liver which scored a DSC of 0.93 and CMD of 0.19 cm. Considering these values as a reference, no statistically different values of DSC were found for heart and contralateral breast, while the remaining six OARs had lower values (Figure 2a). Regarding CMD, only the heart and the ipsilateral kidney were not statistically different from the liver (Figure 2b).

## Discussion

Accurate delineation of tumour volumes and surrounding normal structures represents critical aspects of conformal RT. Several atlases, based on expert consensus, have been published mainly to help the delineation of target volumes [18–22], while potentially critical OARs, such as heart, LAD and brachial plexus have received less attention [23–25], even though their impact is on toxicity. In addition, the contouring process is burdened by time-consuming procedures which may slow the entire workflow. Apart from the introduction of atlas-based auto-contouring programs in the clinical practice, which proved to be beneficial in time reduction compared to the manual contouring [26], the possibility of expanding the role of CSRTs in the pre-planning phase represents a major asset. Several studies have already confirmed the great potential of CSRTs in normal tissue contouring in the head and neck and the pelvic districts [10–13, 27].

This study reported on the variability in delineating chest and upper abdomen OARs by observers from a different background and showed that small variations can be achieved between CSRT and ROs, for all the structures, through adequate contouring training. A 100-hour training course was therefore sufficient to provide the basic contouring skills, but we are aware that continuing contouring workshops are mandatory. Although on the whole, the inter-observer variability was low and all the results were above the thresholds for good concordance, the FRO showed the smallest concordance when compared with the other observers probably due to the short experience in thoracic structures. This observation can strengthen the concept that providing detailed indications might not be sufficient to ensure a consistent way of contouring, particularly in the case of unfavorable patients’ anatomy [26] and clinical practice is crucial to improve skills. In addition, Li *et al* [28] reported that the inter-observer variability might be related to differences in opinion regarding volume boundaries. Efforts to improve contouring consistency and reduce inter-observer variability were successful to fill the gap in education, knowledge and expertise among the observers. In fact, the study showed that a well-trained CSRT acquires site-specific skills of contouring which bring him on the same level of capabilities as those displayed by senior and junior ROs.

Comparing the different OAR delineation, six out of nine OARs showed both parameters above the acceptable threshold and all of them reported at list one parameter above the threshold. The possibility of training CSRTs for helping ROs in the contouring of OARs is appealing, given the increased contouring workload for ROs in the light of the growing complexity of radiation treatment [29]. The evaluation of OAR delineation consistency between CSRT and ROs has been previously studied. Fitzpatrick *et al* [17] evaluated 39 sample prostate plans where the CSRT delineated OARs and concluded that, after proper training, contouring of OARs was successfully carried out by CSRT.

On the other hand, we believe that the CTV delineation remains the responsibility of the ROs, even if some studies addressed the role of CSRT in this field [9, 12, 13]. Batumalai *et al* [9] used the concordance index as an assessment tool to quantify inter-observer variability between four ROs and four CSRTs regarding breast CTV delineation. The analysis revealed high consistency between the different professional figures: the mean concordance index was 0.81 and 0.84 for the RO and CSRT, respectively. Similar results were found in other studies [16, 30].

Regarding the single OAR (Table 2), the LAD presented the largest variability of contouring probably due to the small volume and the difficulty of visualization and identification on the CT imaging, especially without any contrast medium [31]. Similarly, the oesophagus faced the same challenge of being identified along the entire length and presented a borderline DSC. Structures with small volumes are a source of uncertainties and may cause inaccurate dosimetric evaluation, which is exacerbated by highly conformal RT planning techniques such as IMRT and stereotactic RT [6]. For all these reasons, it is recommendable that the RO reviews the contours drawn by the CSRT, especially when dealing with small volumes and complex organs.

The spinal cord presented a good value of DSC (>0.80, Table 2), but a poor CMD (1.22 cm, Table 2) due to the variability in the contouring in cranio-caudal direction. In fact, although the upper and lower limits of the spinal cord contouring were previously established, some observers did not carefully follow the given indications.

This study presented a number of limitations. First, it included only one CSRT and in general a small number of observers. Other studies, like the one conducted by Struikmans *et al* [32], included few observers, but they all were physicians and, therefore, had a common background. Second, only chest and upper abdomen OARs were the objects of investigation, and none of the patients had a complex anatomic conformation which could decrease the adherence to the written indications and the uniformity of contouring [26]. Third, the lack of gold standard OAR delineation may be seen as a limitation [10–12], but the aim of the study was to compare the contouring skills of the CSRT with those of ROs. Other studies on inter-observer variability did not incorporate a gold standard set of contours [7, 28, 33]. Finally, this study did not assess the dosimetric impact on inter-observer variability, although there is a paucity of data on variations in dosimetric planning [28].

## Conclusion

Despite the short training dedicated to learn the contouring of the chest and upper abdomen OARs, the CSRT has achieved a good concordance with the ROs (both JRO and SRO). This study demonstrates that CSRT can successfully accomplish the task of contouring OARs and may represent a reliable resource to enhance departmental planning process efficiencies. Moreover, a good/excellent agreement between ROs and CSRTs in electronic portal imaging (EPI) registration in BC RT [34] and in prostate image-guided RT procedures using cone-beam CT [35] has been documented, encouraging the extension of the CSRT role and their growing involvement in all departmental activities. Further developments will include a larger number of CSRTs and the set up of a multicentric study, in order to increase the robustness of the investigation and at the same time strengthen and validate these preliminary results.

## Authors’ contributions

Eleonora Miglietta is the co-first author. Barbara Alicja Jereczek-Fossa is the co-last author.

## Conflicts of interest

The authors declared that there is no conflict of interest.

## Funding declaration

This study was partially supported by the Associazione Italiana per la Ricerca sul Cancro (AIRC), project IG-14300 ‘Carbon ions boost followed by pelvic photon intensity-modulated RT for high-risk prostate cancer’, registered at ClinicalTrials.gov (NCT02672449), approved by IEO R86/14- IEO 98 and by a research grant from Accuray Inc. entitled ‘Data collection and analysis of Tomotherapy and CyberKnife breast clinical studies, breast physics studies and prostate study’. The Sponsors did not play any role in the study design, collection, analysis and interpretation of data nor in the writing of the manuscript and in the decision to submit the manuscript for publication.

## Figures and Tables

**Table 1.** Mean and SD of DSC and CDM for each couple of observers.

	DSC		CMD (cm)	
Observers couple	Mean	SD	Mean	SD
JRO versus FRO	0.76	0.21	0.84	0.82
FRO versus CSRT	0.76	0.22	0.76	0.69
SRO versus CSRT	0.79	0.18	0.54	0.58
SRO versus JRO	0.80	0.17	0.51	0.53
SRO versus FRO	0.76	0.21	0.75	0.77
JRO versus CSRT	0.83	0.13	0.49	0.43

*JRO, Junior RO; FRO, Fellow RO; CSRT, Clinical specialist radiation therapist; SRO, Senior RO

**Table 2.** Mean and SD of DSC and CMD for each OAR obtain from all observers for all patients.

	DSC		CMD (cm)	
OARs	Mean	SD	Mean	SD
Heart	0.91	0.02	0.23	0.08
Esophagus	**0.66**	0.06	0.89	0.65
Liver	0.93	0.01	0.19	0.13
LAD	**0.34**	0.17	0.90	0.62
Contralateral breast	0.90	0.03	0.55	0.22
Spinal cord	0.80	0.06	**1.22**	1.00
Ipsilateral kidney	0.88	0.04	0.19	0.25
Stomach	0.83	0.09	0.81	0.78
Trachea	0.78	0.06	0.85	0.59

*LAD, Left anterior descendant artery

Poor results (mean DSC < 0.7 and CMD > 1 cm) are in bold
